# Hyaluronic Acid–Alginate Homogeneous Structures with Polylactide Coating Applied in Controlled Antibiotic Release

**DOI:** 10.3390/gels9070526

**Published:** 2023-06-28

**Authors:** Anna Trusek, Maciej Grabowski, Omoyemi Ajayi, Edward Kijak

**Affiliations:** 1 Department of Micro, Nano and Bioprocess Engineering, Wroclaw University of Science and Technology, Wybrzeze Wyspianskiego 27, 50-370 Wroclaw, Poland; maciej.grabowski0520@gmail.com (M.G.);; 2 Department of Dental Prosthetics, Wroclaw Medical University, Krakowska 26, 50-425 Wroclaw, Poland

**Keywords:** hyaluronic acid (HA), polylactide (PLA) coating, core–shell carrier, diffusion drug release, chemical hydrogel crosslinking, Korsmeyer–Peppas model, dental infection

## Abstract

The use of a controlled-release drug carrier is an innovative solution for the treatment of local infections, in particular in dentistry, skin diseases, and in open wounds. The biocompatibility, biodegradability, the possibility of a large amount of drug adsorbed (especially those with hydrophilic properties), and the ability to create structures of any shape and size are the reasons for hydrogels to be frequently studied. The main disadvantage of hydrogel carriers is the rapid rate of drug release; hence, in this study, an attempt was made to additionally chemically cross-link 1-ethyl-3-(3-dimethyl aminopropyl)-1-carbodiimide hydrochloride (EDC) with the hyaluronic acid–alginate (HA–SAL) structure. The answer to significantly reduce the mass flux typical for hydrogel structure was to surround it with a polymer layer using a dry cover. By coating the carriers with polylactide, the release time was increased by around forty times. As the carriers were designed to reduce local bacterial infections, among others in dentistry, the released antibiotics were amoxycillin, metronidazole, and doxycycline.

## 1. Introduction

Hydrogels based on alginate (SAL) and hyaluronic acid (HA) have gained significant attention as drug carriers due to their biocompatibility and biodegradability [1]. Alginate, derived from brown algae, is a non-toxic linear polysaccharide widely used in medical applications, including drug delivery systems, wound dressing, and tissue engineering [2,3]. The gelation of alginate is achieved through the interaction between α-L-guluronate residues and multivalent cations such as Ca^2+^ [4]. Hyaluronic acid, also known as hyaluronan, is a linear mucopolysaccharide composed of glucuronic acid and N-acetylglucosamine units [5]. Traditionally obtained from animal tissues, hyaluronic acid is now predominantly produced through bacterial fermentation and in vitro methods [6,7].

However, in the combination of HA with other components like alginate, gelatin, chitosan, or silk fibroin, hyaluronic acid can form cross-linked hydrogels with improved mechanical properties, controlled-release characteristics, and enhanced homogeneity [8,9,10,11,12,13,14,15,16]. In the pursuit of achieving more uniform and mechanically robust hydrogels, researchers have explored different approaches. Catanzano et al. [9] developed a hyaluronic acid–alginate (HA–SAL) hydrogel using calcium carbonate and gluconic-δ-lactone for gelation initiation. These HA–SAL hydrogels demonstrated excellent handling characteristics and biocompatibility with adipose-derived multipotent adult stem cells and a keratinocyte cell line in vitro [9].

Chemical cross-linking using 1-ethyl-3-(3-dimethyl aminopropyl)-1-carbodiimide hydrochloride (EDC) as the carboxyl-activating agent and adipic dihydrazide (ADH) as the crosslinker has also been explored to create composite hydrogels of HA–SAL [17]. Wang et al. [17] demonstrated that these hydrogels’ mechanical properties and swelling capacity were influenced by the polymer concentration and the HA/SAL molar ratio. These composite hydrogels showed promise for tissue engineering scaffolds and exhibited biocompatibility in vitro and in vivo. Another approach involved using EDC as a cross-linking agent to develop HA–SAL composite hydrogels. Zhang et al. [10] combined oxidized alginate with HA modified with 3,3′-dithiobis(propionohydrazide) and carbodihydrazide to create the composite hydrogel. In vitro, testing demonstrated a sustained release of albumin over 7–8 days, with 50% released within the first 24 h.

Polylactide (PLA), a biocompatible polyester, offers further possibilities in the field of drug delivery [18]. While PLA–HA structures have been explored in biomedical applications [19,20,21], their potential as reservoir carriers or implants still needs to be explored [22,23,24].

Amoxicillin (AMX), a β-lactam antibiotic, demonstrates effective prophylaxis against post-invasive dental procedure bacteremia. AMX-loaded electrospun nanocomposite membranes (PCL/nHAp) exhibit controlled-release profiles over three weeks (drug content: 85–100%), inhibiting bacteria [25,26]. Metronidazole (MZL), a nitroimidazole derivative, is commonly combined with AMX for dental infections [27,28]. In vitro release studies of MZL from dental implants (alginate and alginate/PCL composite rings) show burst release followed by a sustained release for over four weeks. The controlled release of antibacterial agents effectively inhibits *Porphyromonas gingivalis* biofilm formation, achieving 50% release in the first 48 h [29]. MZL is also combined with doxycycline (DXC) in dental treatments [30,31]. Biodegradable implants (polylactide–polyglycolide copolymers) containing MZL and DXC exhibit release rates exceeding the minimum inhibitory concentration of *Escherichia coli* for up to three weeks. DXC-coated nanotube-modified implant surfaces significantly suppress *Porphyromonas gingivalis* growth, showing concentration-dependent antimicrobial activity. The final *P. gingivalis* growth at day 28 is 29.4% lower than the baseline growth [32].

In summary, the synergistic combination of alginate, hyaluronic acid, and polylactide holds immense potential for creating homogeneous structures capable of controlled release of antibiotics. Such structures have not been described in the literature.

This study is mapped out to advance our understanding of incorporating AMX, DXC, and MZL into HA/SAL composite hydrogels. It further seeks to evaluate and compare different methods for achieving stable binding of these antibiotics within the hydrogel matrix and characterize the developed carrier by assessing their drug release profiles and mechanical properties. These comprehensive analyses will provide valuable insights into the potential of these controlled-release systems for efficient antibiotic delivery.

## 2. Results and Discussion

### 2.1. Determination of the Mechanical Strength of Carriers

**Figure 1:**
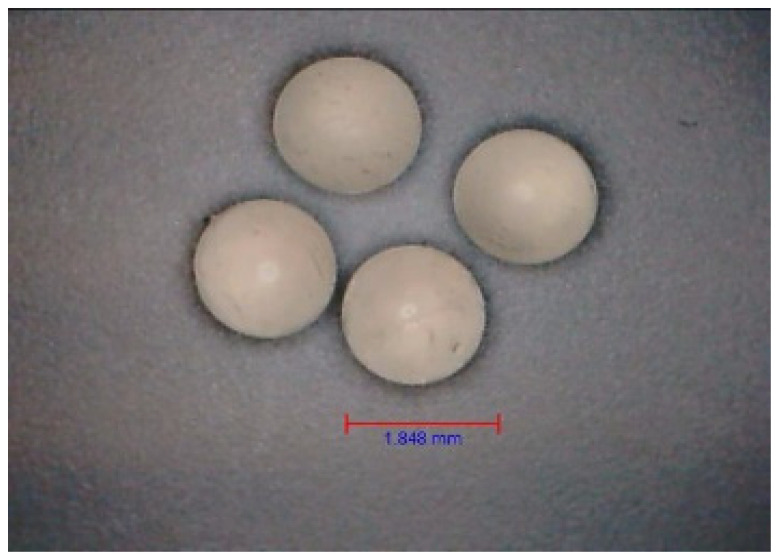
Microscope image of HA–SAL capsules obtained by physical gelation. The head used in the encapsulator had a diameter of 1.5 mm.

The diameter of the carriers was measured to be 1.848 ± 0.116 mm during physical gelation (Figure 1) and 1.733 ± 0.107 mm during crosslinking with EDC. Notably, the diameter of the capsules decreased with longer coating times. Specifically, it measured 1.600 ± 0.086 mm, 1.497 ± 0.082 mm, and 1.345 ± 0.072 mm for coating times of 30, 45, and 60 min, respectively. To assess the strength of the carriers, a dynamometer was used to measure the force required to destroy them. It was observed that the physically gelled carriers exhibited significantly greater strength compared to the chemically gelled structures (Table 1). The observed differences in mechanical strength can be attributed to the distinct gelation mechanisms. During physical gelation, the formation of a three-dimensional network structure occurs, leading to stronger and more resilient carriers [17]. On the other hand, chemical gelation, facilitated by EDC cross-linking, may result in a less robust network structure, leading to decreased mechanical strength.

Also, when subjected to external forces, the capsules from the CaCl_2_ baths tended to flatten before breaking, whereas the capsules from the second CaCl_2_ bath with EDC cross-linking broke without significant deformation. The compressive force required to break the physically gelled carriers was approximately three times greater than that of the chemically gelled capsules. Interestingly, the introduction of PLA coating further enhanced the compressive force needed to break the carriers, resulting in an additional three-fold increase, as demonstrated in Table 1. Hence, the enhanced performance of physically gelled carriers, coupled with the additional reinforcement provided by the PLA coating, demonstrates its potential for applications where durability and resistance to external forces are crucial.

### 2.2. Mass Transport from HA–SAL Homogeneus Carriers

**Figure 2:**
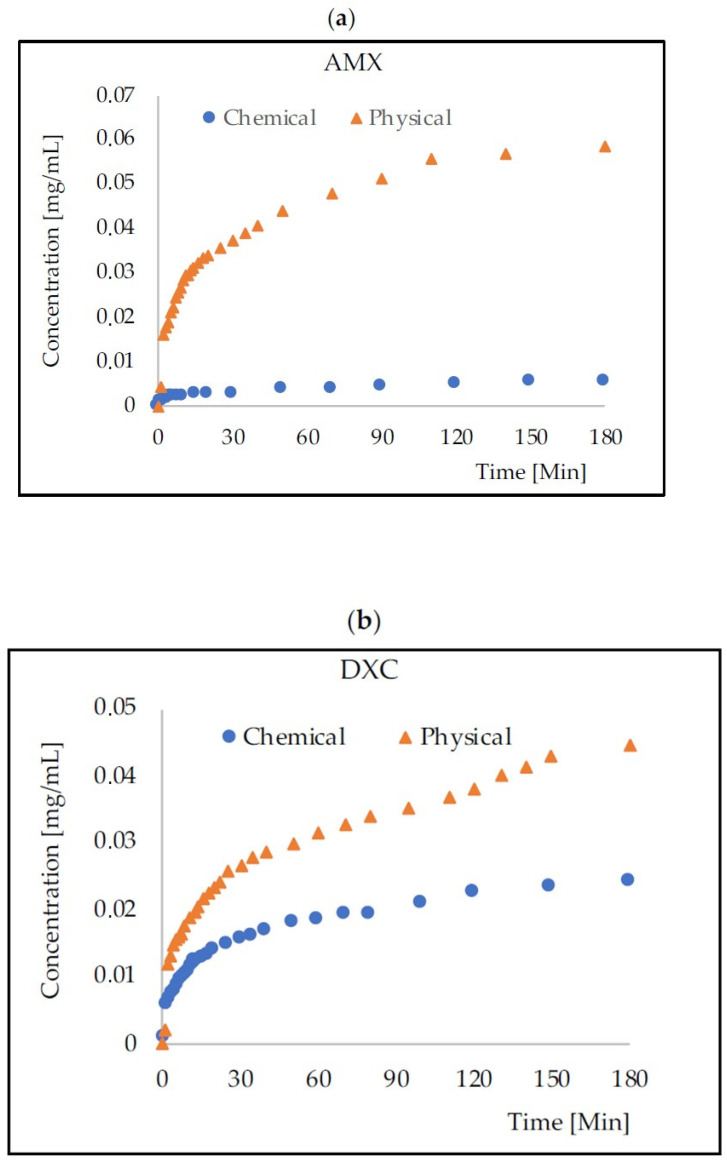

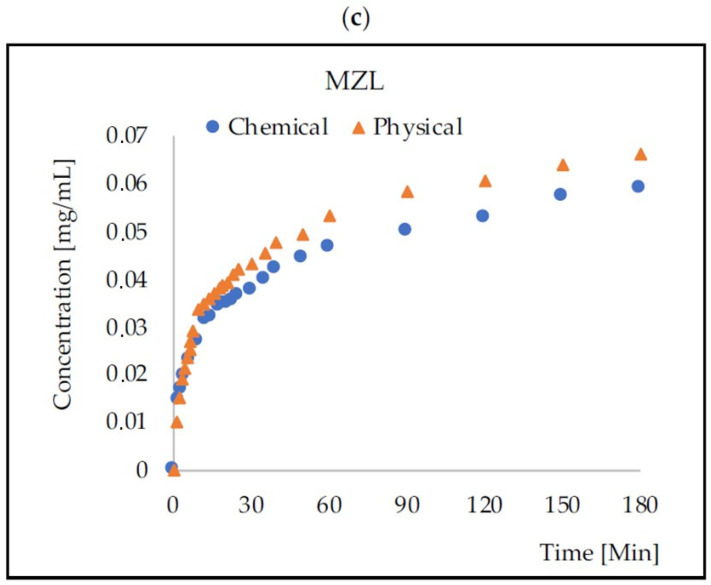
Change in AMX (**a**), DXC (**b**), and MZL (**c**) concentrations over time at 37 °C (averaged values from three series). Capsule volume to receiver solution volume was 1:9.47; capsules diameter was 1.748 ± 0.107 mm. The initial drug concentration inside the capsules was 1 mg/mL.

The rate of release of antibiotics from the tested physically and chemically cross-linked carriers was monitored spectrophotometrically in the receiving solution (Figure 2). Table 2 presents the percentages of the released antibiotic mass after 3, 10, and 24 h of monitoring.

**Figure 3:**
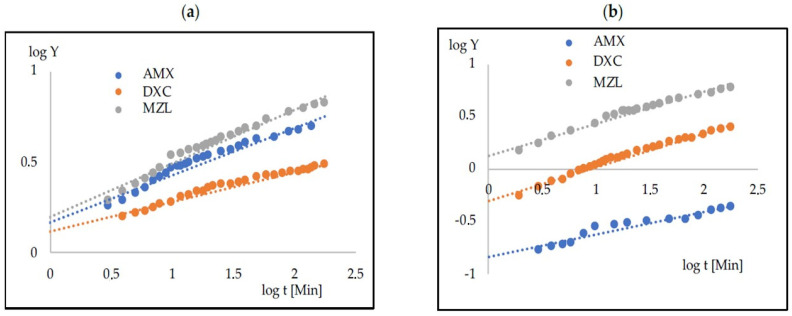
Fitting the experimental points to the Korsmeyer–Peppas model for the carriers that are physically (**a**) and chemically (**b**) gelled for AMX, DXC and MZL.

The experimental data collected within the initial 180 min of the process were subjected to fitting with a diffusion-based release model. Among the considered models, the Korsmeyer–Peppas model [33] (Equation (1)) demonstrated the good agreement (R^2^ > 0.968) in all cases—Figure 3. The determined coefficients corresponding to this model are provided in Table 3.

### 2.3. Coating HA–SAL Carriers with PLA

**Figure 4:**
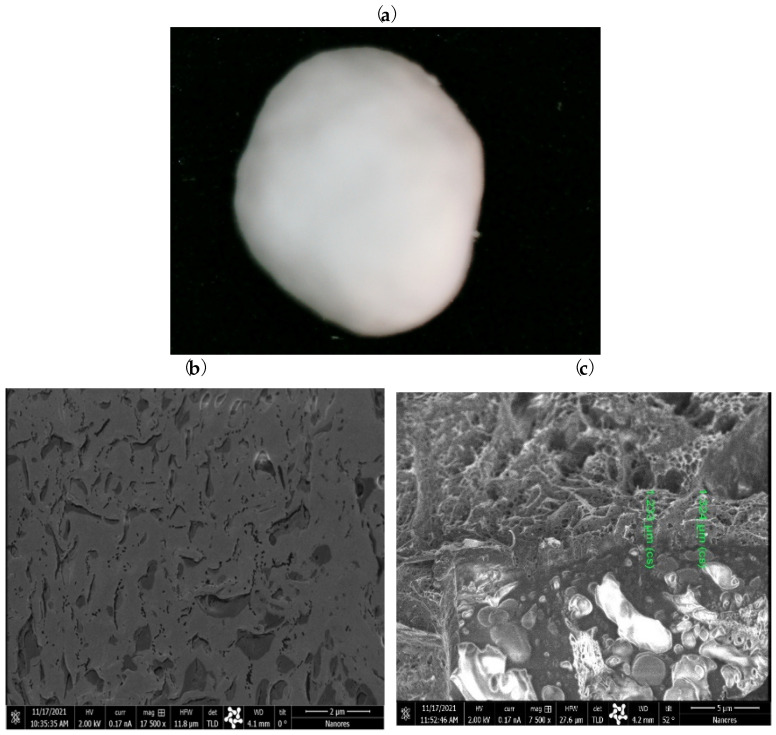
Carriers covered with PLA layer-coated hydrogel capsules (2.5% polylactide, 60 min, 45 °C). Microscopic image (**a**), SEM surface image (**b**), and SEM/Ga-FIB section image (**c**).

Under optimized conditions (2.5% PLA, 60 min), a homogeneous coating was successfully achieved (Figure 4), resulting in a reduced diameter of 1.345 ± 0.072 mm. The comprehensive analysis of SEM/Ga-FIB images highlighted the critical role of the coating process time in obtaining a uniform layer. Varying the coating time revealed distinct differences in the uniformity of the HA–SAL capsule coatings. Shorter coating times resulted in uneven and non-uniform coatings, while longer coating times improved homogeneity. These findings emphasize the importance of optimizing the coating process time to achieve consistent and uniform coatings, which are essential for the carriers’ performance. The resulting layer thickness ranged between 1.21–1.33 um.

### 2.4. Mass Transport from PLA–HA–SAL Carriers

**Figure 5:**
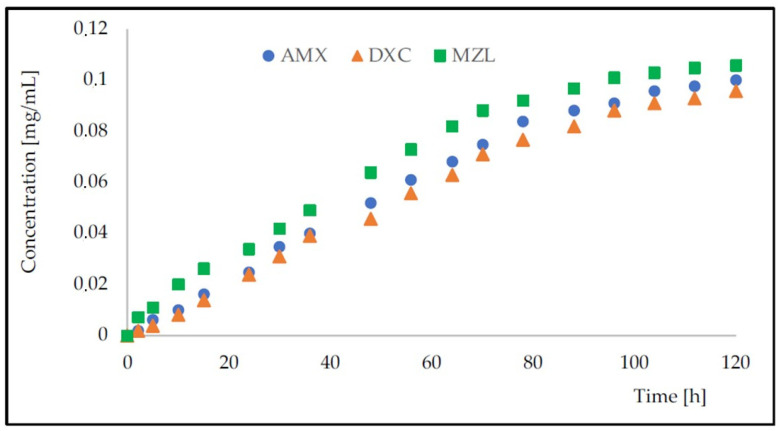
Change in AMX, DXC, and MZL concentrations over time at 37 °C (averaged values from three series) for HA–SAL carriers with PLA coating. Capsule volume to receiver solution volume was 1:9.47; capsules diameter was 1.345 ± 0.072 mm. The initial drug concentration inside the capsules was 1 mg/mL.

**Figure 6:**
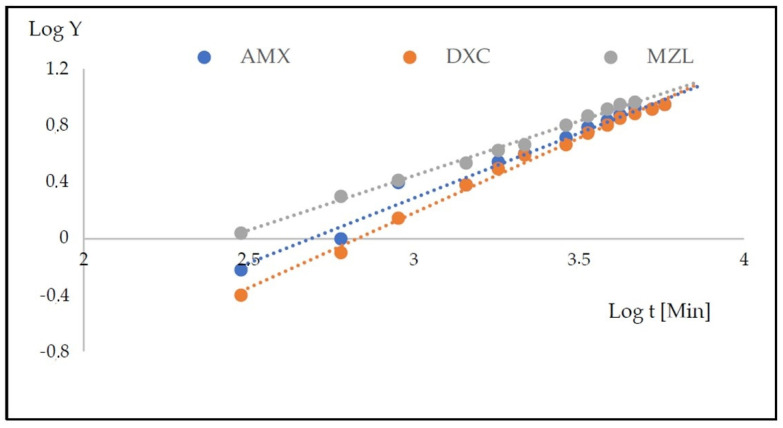
Fitting the experimental points to the Korsmeyer–Peppas model for the carriers with PLA coating for AMX, DXC and MZL.

The rate of release of antibiotics from the structures based on HA–SAL hydrogel physically gelled and with PLA coating was monitored spectrophotometrically in the receiving solution over five days (Figure 5). Table 4 presents the calculated values of the Korsmeyer–Peppas model [33] and Figure 6 the fitting of the points.

## 3. Conclusions

As a natural component in the body, HA is a particularly noteworthy hydrogel. The research presented in the paper showed that HA-based composite structures could be used for drug dosing. Both physical and chemical gelling were analyzed. Physical gelling was based on CaCO_3_-GDL, while an addition of GDL to cross-linking baths slows release of calcium ions to obtain homogeneously cross-linked hydrogels. Capsules obtained by this method were characterized by the expected homogeneity, i.e., 100% encapsulation efficiency and 100% possibility of releasing the encapsulated drug molecules.

Chemical cross-linking of HA and SAL was based on activating their carboxyl groups with EDC and then linking the chains through the ADH linker. The method of packing drugs into such a structure turned out to be effective in the case of antibiotics that do not contain carboxyl or amino groups (in the case studied, for metronidazole). Especially AMX containing the carboxyl group was permanently bound to the network made of HA and SAL and consequently released to a very low degree (14.4% after 24 h).

Capsules (carriers) obtained by chemical cross-linking were characterized by significantly lower mechanical resistance. This feature and the inability to release all encapsulated drug molecules in the case of amoxicillin and doxycycline resulted in the choice of physically cross-linked capsules for the production of core–shell structures. The premise for producing hydrogel structures with a polylactide coating was a very fast transport of drugs from homogeneous HA–SAL structures.

The obtained PLA–HA–SAL carriers were characterized by a relatively uniform coating layer thickness and a slow release of drugs. This issue still needs to be elaborated to optimize the coating conditions using a Dry-Coater, influencing the thickness of the layer and the transport speed. Using a polylactide layer reduced the transport rate, but long-term mass transport was achieved lesser than with enzyme-controlled carriers [34].

The rate of drug release from both homogeneous HA–SAL structures and the PLA-HA–SAL core–shell was described using the Korsmeyer–Peppas model. The coefficients obtained for HA–SAL are similar to those reported in the literature for other hydrogels [35,36]. In the case of PLA-HA–SAL carriers, the value of the n coefficient for larger drug molecules (M_w_ in the range 350–450 g/mol) is close to unity, which indicates the release rate consistent with the first-order kinetics. This as well as the lower values of the k coefficient show that the polylactide layer generates a significant resistance in mass transport [37]. Considering the components used in the proposed carrier, it is fully biocompatible [38,39].

In combination with alginate, both physically and chemically gelled hyaluronic acid can form a biocompatible matrix (HA–SAL) for packing a mass of drugs. However, without an additional polymer layer, e.g., made of PLA, drug release is fast and ends after several hours. The HA–SAL structure with PLA coating layer was developed for long-term drug carriers used, for example, in inflammatory conditions in dentistry. Further research will focus on developing the relationship between the thickness of the polylactide layer and the rate of drug release and thus the duration of treatment.

## 4. Materials and Methods

### 4.1. Materials

**Figure 7:**
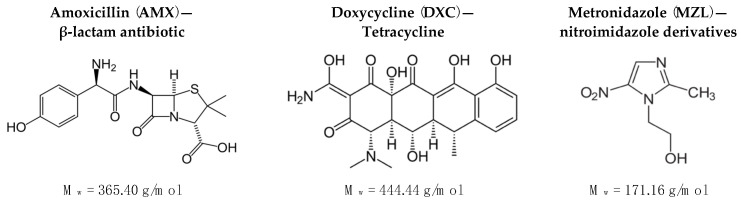
Antibiotics used in the research (M_w_—molecular weight).

Sodium Alginate (SAL, M.w. 216 g/mol), Hyaluronic acid (HA, M.w. 8–15 kDa), Poly (D,L-lactide) (PLA, M.w. 75–120 kDa), N-(3-Dimethylaminopropyl)-N′-ethylcarbodiimide hydrochloride (EDC), Amoxicillin (AMX), Metronidazole (MZL), and Doxycycline (DXC) were provided by Sigma-Aldrich (St. Louis, MO, USA). The structures and molecular weights are presented in Figure 7. 

Adipic dihydrazide (ADH), D-(+)–Gluconic acid δ-lactone (GDL), 4-Morpholineethanesulfonic acid, 2-(N-Morpholino)ethanesulfonic acid sodium salt (MES), and Ringer tablets were manufactured by Merck (Darmstadt, Germany). CaCl_2_ was provided by Pol-Aura (Dywity, Poland).

### 4.2. Equipment

For the preparation of the carriers, B-390 encapsulator (Buchi, Flawil, Switzerland) and Caleva’s dry cover (Dorset, UK) were used.

Electronic caliper 150 × 0.01 mm (Asimeto, Weißbach, Germany), Camera Microscope Digimicro Lab. 5.0 (Toolcraft, Georgensgmünd, Germany), SEM/Ga-FIB FEI Helios NanoLab™ 600i (FEI, Thermo Fisher Scientific, Eindhoven, The Netherlands), and eZT dynamometer (Imada, Aichi, Japan) were used to carriers characterization.

UV–VIS Spectrophotometer UV-1280 (Shimadzu, Kioto, Japan) was applied in the drug concentration determination.

### 4.3. HA–SAL Composite Carrier Preparation by Physical Gelation

The procedure developed by the team of O. Catanzano [9] was used. The temperature was kept at 40 °C and the stirring at 140 rpm. A 1% *w*/*v* SAL solution was prepared by dissolving SAL in distilled water. GDL was added to the SAL solution so that its concentration would be 64 mM. After that, HA was added in an amount corresponding to 25% of the weight of the SAL previously added. An antibiotic (AMX, DXC, or MZL) was added to the resulting mixture to receive its 1 g/L concentration. The solution was dripped into a bath containing 10% CaCl_2_ *w*/*v* and the given antibiotic at a 1 g/L concentration using an encapsulator. The capsules were cross-linked for 24 h at 4 °C (without stirring).

### 4.4. HA–SAL Composite Carrier Preparation by Chemical Gelation

The procedure was based on the method described by M.-D. Wang [17]. The temperature was kept at 40 °C and the stirring at 140 rpm. MES buffer (100 mM) was prepared and adjusted to pH 5.0 with NaOH. In this buffer, a solution containing 3.0% *w*/*v* SAL and a solution with 0.75% *w*/*v* HA and ADH at a concentration of 0.216 mmol/L were prepared. Both solutions were mixed in a 1:1 ratio; thus, the ratio HA:SAL was 1:4. Antibiotic (AMX, DXC, or MZL) was added to the resulting mixture to obtain a concentration of 1 g/L. Cross-linking bath consisted of 10% *w*/*v* CaCl_2_ and 0.05 M EDC and a given antibiotic with a concentration of 1 g/L. The prepared HA/SAL solution was added dropwise to the cross-linking bath using an encapsulator and then placed in a refrigerator for 24 h.

### 4.5. Coating HA–SAL Carriers with PLA

Tests were carried out with the coating of HA–SAL spherical structures (prepared by physical gelation) with polylactic (PLA) using a dry cover. PLA solution with a concentration of 2.5% *w*/*v* was prepared in methylene chloride. The process temperature was 45 °C. The solution was dosed at a speed of 4 RPM/min, air flow was 16 m/s, shaking at 21.2 hZ, and pressure at the nozzle of 0.5 bar. Capsule coating was carried out for 30–60 min.

**Figure 8:**
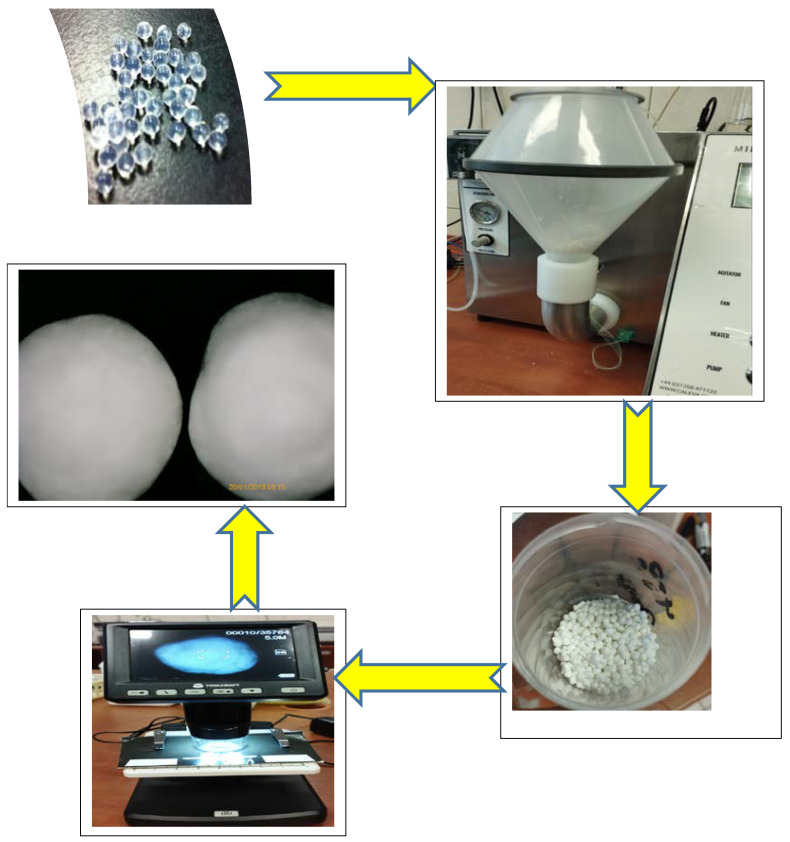
The procedure for coating capsules (PLA–HA–SAL).

The procedure of the coating of capsules is presented in Figure 8.

### 4.6. Characteristics of the Obtained Carriers

Capsule (carrier) diameters were measured with an electronic caliper. Microscopic pictures were taken using camera microscope. A two-beam microscope SEM/Ga-FIB was used to investigate the structure of PLA layer coating the capsules. The microscope comprises ultrahigh-resolution electron and ion microscopy. The energy-focused beam of gallium ions allows the selective removal of the preparation material and modification at the nanoscale, such as the sample cross sections. Before the analyses, the samples were coated with gold. The mechanical strength of the carriers was determined using a dynamometer. Ten randomly selected carriers were tested from each series.

### 4.7. Mass Transport Monitoring

The antibiotic release rate was tested according to the following procedure. A known (4.75 mL) volume of capsules was measured and then rinsed with distilled water. The capsules were transferred to a thermostated (37 °C) stirred tank containing 45 mL of Ringer’s fluid. The ratio of the volume of the capsules to the solution was 1:9.47. The mass of antibiotics released over time was determined by measuring the absorbance using UV–VIS Spectrophotometer at a wavelength chosen for each substance. The concentration was read from the absorbance values using the standard curve equation (Table 5).

The Korsmeyer–Peppas model [33] (Equation (1)) was used to describe the mass release. The least squares method was used in the fitting of the coefficient values determination.

Log Y = log k + n·log t(1)

where:

Y = m_t_/m_0_·100 [%]
m_0_, m_t_—initial and released mass [mg]
t—time [Min]
k—coefficient
n—coefficient [1/Min]

## Figures and Tables

**Table 1:** Pressure force [N] (average of ten measurements) required to destroy monolithic HA–SAL composite carriers obtained by physical or chemical crosslinking and core–shell HA–SAL carriers obtained by PLA-coated method after physical crosslinking. Before testing, the carriers were stored in Ringer’s solution at 37 °C for 24 h.

Antibiotic	AMX		DXC		MZL	
	Physical	Chemical	Physical	Chemical	Physical	Chemical
Pressure force [N]	1.490 ± 0.125	0.425 ± 0.075	1.625 ± 0.175	0.600 ± 0.05	1.675 ± 0.15	0.625 ± 0.05
	PLA-coated core–shell carriers					
Pressure force [N]	4.625 ± 0.125		4.525 ± 0.125		4.765 ± 0.175	

**Table 2:** Percentage of the antibiotic mass after 3, 10, and 24 h of the release process.

Antibiotic	AMX		DXC		MZL	
	Physical	Chemical	Physical	Chemical	Physical	Chemical
Time [h]	-	-	-	-	-	-
1	21.3%	1.8%	14.8%	9.1%	29.9%	17.8%
2	38.7%	3.7%	27.7%	16.5%	50.1%	41.1%
3	55.7%	5.3%	44.2%	22.9%	62.6%	55.9%
5	82.2%	7.8%	72.2%	30.2%	81.0%	78.2%
10	90.7%	9.7%	88.0%	37.8%	96.7%	91.1%
24	100.0%	14.4%	100.0%	55.3%	100.0%	100.0%

**Table 3:** Coefficients (averaged values from three series) of the Korsmeyer–Peppas model antibiotics released from HA–SAL carriers that are physically and chemically gelled.

Antibiotic	AMX		DXC		MZL	
	Physical	Chemical	Physical	Chemical	Physical	Chemical
n [1/Min]	0.259	0.212	0.167	0.318	0.296	0.300
k [-]	1.491	0.146	1.303	1.995	1.562	1.348
R^2^	0.956	0.925	0.976	0.983	0.972	0.978

**Table 4:** Coefficients (averaged values from three series) of the Korsmeyer–Peppas model for antibiotics released from PLA–HA–SAL carriers.

Antibiotic	AMX	DXC	MZL
n [1/Min]	0.922	1.061	0.774
k [-]	0.003	0.001	0.013
R^2^	0.976	0.995	0.994

**Table 5:** Spectrophotometric measurement of the concentration of individual antibiotics.

Antibiotic	AMX	DXC	MZL
Wavelength [nm]	274	367	320
Standard curve	A = 2.67 C [mg/mL]	A = 28.283 C [mg/mL]	A = 54.508 C [mg/mL]
